# Case Report: Prostate adenocarcinoma with peritoneal carcinomatosis and elevated CEA mimicking colorectal cancer: a diagnostic dilemma

**DOI:** 10.3389/fonc.2026.1736081

**Published:** 2026-04-01

**Authors:** Hala Majeed, Nathan Shelman, Jitesh A. Patel, Janeesh S. Veedu, Insija I. Selene, Ashlyn Whitesell, Zin W. Myint

**Affiliations:** 1 Department of Medicine, Division of Medical Oncology, Markey Cancer Center, University of Kentucky, Lexington, KY, United States; 2 Department of Pathology and Laboratory Medicine, University of Kentucky, Lexington, KY, United States; 3 Department of Surgery, Division of Colorectal Surgery, Markey Cancer Center, University of Kentucky, Lexington, KY, United States; 4 College of Pharmacy, Markey Cancer Center, University of Kentucky, Lexington, KY, United States

**Keywords:** elevated CEA, low PSA levels, peritoneal carcinomatosis, prostate adenocarcinoma, rectal obstruction

## Abstract

This case report describes an exceptionally rare manifestation of prostate adenocarcinoma presenting with peritoneal carcinomatosis, rectal obstruction, and markedly elevated carcinoembryonic antigen (CEA), closely mimicking colorectal cancer. A 65-year-old man with localized high-grade prostate adenocarcinoma (Gleason 4 + 4) underwent robotic prostatectomy in November 2020, followed by salvage radiation and stereotactic body radiation therapy for early biochemical recurrence. He achieved an excellent response to androgen deprivation therapy (ADT) combined with abiraterone and prednisone, maintaining an undetectable prostate-specific antigen (PSA) level for over 2 years. In late 2024, the patient developed hematochezia, narrow stools, and abdominal pain. Imaging revealed a rectal mass, diffuse peritoneal carcinomatosis, and new hepatic lesions. Despite an undetectable PSA, his serum CEA exceeded 7,000 ng/mL, strongly suggesting a colorectal primary. Multiple biopsies and immunohistochemical stains were inconclusive, showing poorly differentiated adenocarcinoma without definitive prostate or colorectal markers. Given the clinical presentation, FOLFOX chemotherapy was initiated empirically for presumed colorectal carcinoma. Next-generation sequencing (NGS) of rectal tissue ultimately identified a TMPRSS2::ERG fusion identical to that in the original prostatectomy specimen, strongly supporting a diagnosis of metastatic prostate adenocarcinoma origin. A subsequent liver biopsy corroborated the findings. The patient transitioned to docetaxel plus ADT, resulting in symptomatic improvement and partial radiologic response. Despite temporary stabilization, progressive bowel dysfunction required a palliative colostomy. Omental biopsies again confirmed metastatic prostate adenocarcinoma. This report underscores several key clinical lessons. First, peritoneal metastasis from prostate cancer is exceedingly uncommon and can masquerade as gastrointestinal malignancy, particularly when accompanied by high CEA levels and rectal involvement. Second, standard markers such as PSA and immunohistochemistry may be misleading in atypical presentations. Finally, the case highlights the decisive role of molecular diagnostics, specifically NGS, in identifying tumor origin and preventing misdiagnosis.

## Introduction

Prostate cancer is the most diagnosed malignancy among men and a leading cause of cancer-related death (1). While it frequently metastasizes to the bones, lymph nodes, liver, and lungs through hematogenous and lymphatic dissemination, peritoneal carcinomatosis and direct rectal involvement are exceedingly rare (2, 3). These atypical metastatic patterns often mimic primary gastrointestinal malignancies (4), especially when clinical features such as hematochezia, bowel obstruction, and elevated carcinoembryonic antigen (CEA) levels are present.

Serum prostate-specific antigen (PSA) is a well-established biomarker for detecting and monitoring prostate cancer (5); however, its utility may be limited in rare presentations where PSA remains undetectable despite widespread disease. Advances in diagnostic imaging, particularly prostate-specific membrane antigen positron emission tomography (PSMA PET), which received U.S. Food and Drug Administration (FDA) approval in 2020 (6), and molecular diagnostics, such as next-generation sequencing (NGS), have enhanced our ability to identify occult or atypical metastatic disease.

Herein, we present a diagnostically challenging case of metastatic prostate cancer presenting as a rectal mass with associated peritoneal carcinomatosis and liver metastases in the setting of an undetectable PSA and markedly elevated CEA. This case underscores the importance of maintaining a broad differential diagnosis and highlights the role of advanced molecular and imaging tools in identifying rare patterns of metastatic spread.

## Case description

A 65-year-old man underwent robotic prostatectomy for localized prostate adenocarcinoma, Gleason group 8 (4 + 4), in November 2020. He developed biochemical recurrence in September 2021, confirmed by CT imaging that demonstrated borderline enlarged left external iliac nodes. He subsequently received salvage radiation to the prostate bed and stereotactic body radiation therapy (SBRT) to the left external iliac node. By April 2022, his PSA had increased to 12 ng/mL, and PSMA PET revealed recurrence in the prostate fossa, multiple pelvic and para-aortic lymph nodes, the right iliac crest, and the left proximal humerus. He was started on androgen deprivation therapy (Lupron) with abiraterone and prednisone, which reduced his PSA to <0.01 ng/mL. Despite a durable response, in October 2024, his PSA rose slightly to 0.03 ng/mL with progression seen on PSMA PET. After SBRT to the iliac bone and para-aortic nodes, he developed hematochezia, narrow stools, abdominal pain, and constipation in November 2024, raising concern for a colorectal malignancy.

A dedicated pelvic MRI demonstrated an annular, circumferential rectal mass measuring 8.3 cm in craniocaudal length with marked wall thickening up to 18 mm and luminal narrowing. The lesion was located in the lower rectum (2 cm from the anal verge) and was classified as MR-T4b, with invasion of the internal anal sphincter and possible involvement of adjacent structures, including the bulbar urethra. Extramural vascular invasion was present, and mesorectal lymph nodes demonstrated morphologic features concerning nodal metastasis. The tumor straddled the anterior peritoneal reflection. These imaging features were radiographically characteristic of a locally advanced primary rectal adenocarcinoma. He was subsequently referred to our surgical oncology where a flexible sigmoidoscopy in December 2024 was performed, which demonstrated moderately localized, edematous, erythematous, and friable mucosa in the mid and distal rectum. Biopsy showed a poorly differentiated adenocarcinoma, which appeared to be beneath the overlying non-dysplastic colorectal mucosa. The tumor demonstrated predominantly submucosal growth beneath intact, non-dysplastic rectal mucosa, a pattern more typical of secondary involvement than primary colorectal adenocarcinoma. Multiple immunostains were performed to classify the tumor cells. However, the amount of tumor was scant in nature and hence may not be representative of the entire process.

A third biopsy in January 2025 was obtained, which revealed a rectal mass with scant presence of poorly differentiated adenocarcinoma in the submucosa. Multiple immunostains were performed. The tumor cells stained diffusely positive for CK7 and showed focal NKX3.1 nuclear immunoreactivity, which in this clinical context is considered highly sensitive and specific for prostatic origin.

The patient’s PSA remained undetectable; however, CEA was markedly elevated at 5,496 ng/mL, a level more commonly associated with colorectal cancer, although rare cases of aggressive metastatic prostate carcinoma have been reported to produce markedly elevated CEA. Subsequent CT imaging in February 2025 showed diffuse omental nodularity and hepatic lesions, further raising suspicion for a colorectal primary. Pathology remained inconclusive on multiple biopsies, underscoring the diagnostic challenge. Despite undetectable PSA, his CEA continued to rise, exceeding 7,331 ng/mL, leading to discontinuation of abiraterone and initiation of FOLFOX. This was later revised when NGS of the rectal lesion confirmed prostatic origin, identifying a TMPRSS2::ERG fusion concordant with the original prostatectomy specimen.

Given the clinical suspicion of primary colorectal cancer, he was started on Cycle 1 of FOLFOX. However, NGS results from the rectal tissue identified a TMPRSS2::ERG fusion also seen in the original prostatectomy specimen, supporting a clonal relationship between the two lesions, strongly supporting that the rectal mass represented metastatic prostatic adenocarcinoma. No high-grade neuroendocrine features were present. He also underwent a liver biopsy, and pathology showed adenocarcinoma consistent with prostatic origin. A baseline PSMA PET/CT was obtained, which showed diffuse, multinodular, hypermetabolic activity along the anterior peritoneal margin and upper abdomen, particularly around the liver, consistent with peritoneal carcinomatosis.

The findings indicated diffuse peritoneal metastatic disease, hypermetabolic rectal metastasis, and hepatic involvement likely due to a combination of peritoneal and hematogenous spread.

**Figure 1:**
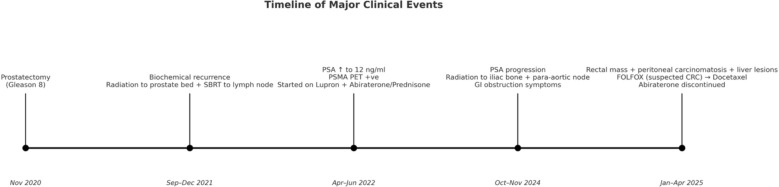
Timeline of clinical events and presentation.

**Figure 2:**
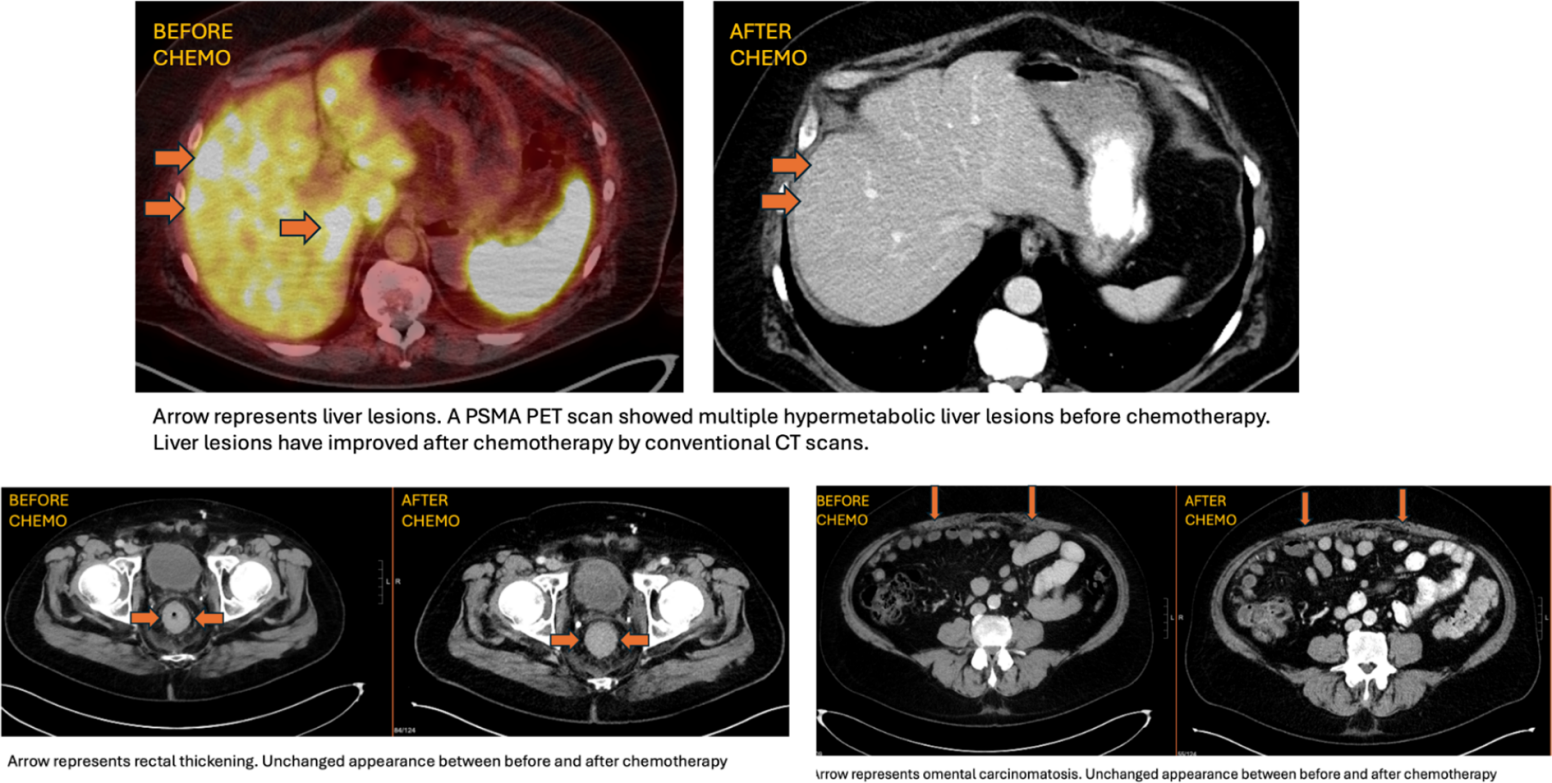
Treatment response comparing before docetaxel and five cycles after docetaxel.

**Figure 3:**
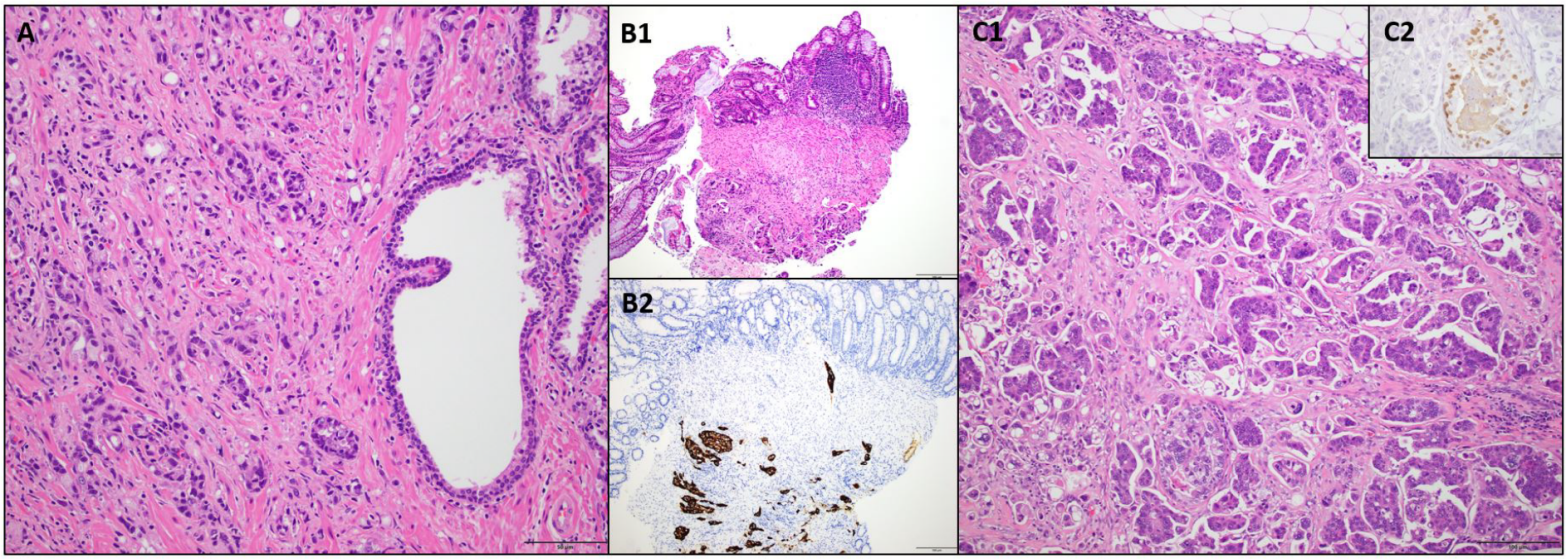
Histopathology images. **(A)** Primary prostatic adenocarcinoma (prostatectomy, 2020), scored as Gleason 4 + 5. Infiltrating tumor cells at left, benign prostatic glands at right (H&E, ×200). **(B1)** Rectum (biopsy, 2025). Infiltrating tumor cells within submucosa (bottom center) and overlying benign rectal epithelium (top, left) (H&E, ×100). **(B2)** Infiltrating tumor cells within submucosa immunoreactive (brown stain) and overlying benign rectal epithelium non-reactive (top, left) (CK7, ×100). **(C1)** Omentum (resection, 2025). Infiltrating tumor cells within a fibrous stroma. Benign adipose tissue at top center (H&E, ×100). [**(C2)**, inset] High magnification of infiltrating tumor cells within omentum with focal (nuclear) immunoreactivity for prostate-specific marker (brown stain) (NKX3.1, ×400).

FOLFOX was stopped, and the patient was given the first cycle of docetaxel along with Lupron in February 2025. **Figure 1** shows a timeline of a clinical event. His initial symptoms of hematochezia and constipation improved following prompt chemotherapy. Although colectomy had been planned for his obstructive symptoms, the procedure was deferred because of symptomatic improvement. PSA remained undetectable. He had restaging scans after four cycles of docetaxel, which showed improvement in hepatic lesions with stable peritoneal carcinomatosis (**Figure 2**). However, the patient developed worsening fecal incontinence with a significant decline in quality of life. In September 2025, he underwent a palliative colostomy for symptomatic relief. Intraoperatively, the omentum was noted to be bulky with metastatic implants and partial small bowel obstruction secondary to peritoneal disease. Pathologic evaluation of the omental specimen confirmed metastatic prostate adenocarcinoma (**Figure 3**).

## Patient perspective

The patient shared that his experience was marked by confusion and anxiety due to the unexpected recurrence and the uncertainty of the diagnosis. The development of hematochezia and bowel obstruction, coupled with markedly elevated CEA levels, led him to believe he had developed a new colorectal cancer. He described this period as particularly distressing, especially when multiple biopsies failed to clarify the diagnosis. The eventual confirmation that the disease represented a recurrence of prostate cancer, rather than a second primary malignancy, brought mixed feelings of relief and concern. He expressed appreciation for the persistence of his medical team in pursuing advanced diagnostic testing, including next-generation sequencing, which ultimately clarified the origin of the cancer and guided appropriate therapy.

Following chemotherapy, he experienced meaningful symptom relief, which he described as a turning point in his journey. However, over time, progressive bowel dysfunction and fecal incontinence began to affect his quality of life. He later underwent a palliative colostomy, which provided comfort and allowed better symptom control. Despite the ongoing challenges of advanced disease, he continues to approach each day with optimism and gratitude, maintaining a positive attitude and appreciation for the care and support he receives.

## Discussion

Peritoneal metastasis in prostate cancer is an uncommon and diagnostically challenging manifestation, typically observed in patients with long-standing, high-grade disease. Although peritoneal carcinomatosis is far more commonly associated with gastrointestinal malignancies, particularly colorectal adenocarcinoma, rare cases of peritoneal dissemination from prostate adenocarcinoma have been reported (7–10). A literature search was performed using PubMed, limited to case reports involving human subjects published within the past 10 years. The search terms used were “prostate cancer” AND “peritoneal metastasis”, and only full-text articles were considered. This search yielded 10 results, of which eight full-text articles were accessible. Upon reviewing these articles, seven were deemed relevant to our study. Among these, only four case reports described peritoneal metastasis occurring specifically after surgical intervention for prostate cancer.

A review of six published cases (7–10) (**Table 1**) revealed that all patients had previously undergone radical prostatectomy and most had received salvage therapies such as androgen deprivation therapy (ADT), radiotherapy, or chemotherapy before developing peritoneal spread. Peritoneal involvement was often discovered incidentally during surgical procedures or through advanced imaging conducted for unrelated concerns. Confirmatory diagnosis required histopathologic evaluation, often via biopsy during exploratory laparoscopy or laparotomy. Imaging modalities included pelvic MRI, ^18^F-choline PET, and PSMA-based PET scans, with one case demonstrating the added value of a novel [^89^Zr]Zr-PSMA-617 tracer (8) when conventional imaging was inconclusive.

**Table 1:** Summary of clinical presentation, diagnostic workup, and treatment modalities in patients with recurrent disease involving peritoneal metastases following radical prostatectomy for prostate cancer, based on a review of the literature.

Case	Patient presentation	Diagnostic methods	Treatment
1.	72-year-old man with Gleason 3 + 3 prostate cancer (radical prostatectomy in 2004); rising prostate-specific antigen (PSA) since 2010. During planned inguinal surgery in 2016, exploratory laparoscopy revealed multiple small peritoneal nodules without ascites (7).	Pelvic multiparametric MRI, ^18^F-choline PET–MRI, PET–CT, ^68^Ga-PSMA PET/CT, and exploratory laparoscopy. Histopathology confirmed carcinoma (7).	Androgen deprivation therapy (ADT) with degarelix and stereotactic body radiotherapy (SBRT) (7).
2.	57-year-old man with Gleason 4 + 3 prostate cancer treated with radical prostatectomy in 2005 with persistent postoperative PSA, prompting early salvage radiotherapy. In 2018, planned surgery for suspected cholangiocarcinoma incidentally revealed peritoneal nodules (7).	MRI, ^18^F-choline PET-CT, bone scan, ^68^ Ga-PSMA PET-CT, abdominal CT-scan with cholangio-MRI. Biopsy and histopathology confirmed results (7).	ADT + SBRT + enzalutamide (7).
3.	60-year-old man with Gleason 4 + 4 prostate cancer underwent radical prostatectomy in 2005, followed by salvage ADT and radiotherapy due to early biochemical recurrence. PSA rise in 2016 led to imaging suggesting liver lesion, but exploratory laparoscopy revealed multiple peritoneal metastases (7)	Pelvic MRI, ^68^Ga-NODAGA-MJ9, ^18^F-choline PET-CT, ^68^Ga-PSMA PET-MRI, and a dedicated MRI. Biopsy from exploratory laparotomy confirmed diagnosis (7).	ADT, radiation therapy, surgical resection of suspicious nodules, and docetaxel (7).
4.	64-year-old man with prior radical prostatectomy and rising PSA despite multiple negative findings on conventional [^68^Ga]Ga-PSMA-11 and [^18^F]FDG PET/CT scans. To localize the source of recurrence, a novel PSMA tracer, [^89^Zr]Zr-PSMA-617, was used, which successfully revealed both local recurrence and diffuse peritoneal carcinomatosis—findings previously undetected with standard imaging techniques (8).	[^68^Ga]Ga-PSMA-11, [^18^F]FDG PET/CT scans, and PET with novel PSMA tracer, [^89^Zr]Zr-PSMA-617. Biopsy from peritoneal lesions confirmed metastatic prostate adenocarcinoma (8).	NA
5.	68-year-old man with Gleason 5 + 4 prostate cancer. Four years post-radical prostatectomy presented with weight loss, abdominal pain, and a markedly elevated PSA of 104 ng/mL. Imaging revealed local recurrence along with multiple nodular peritoneal lesions and abdominopelvic lymphadenopathy (9).	^68^Ga prostate-specific membrane antigen (PSMA) PET/CT for restaging, biopsy, and histopathology for confirmation (9).	Docetaxel (9)
6.	58-year-old man with Gleason 4 + 4 prostate cancer. Eleven years after radical prostatectomy and salvage radiotherapy, PSA rose to 30 ng/mL. PSMA PET revealed falciform ligament lesions, and exploratory laparoscopy confirmed disseminated peritoneal carcinomatosis (10).	^68^Ga prostate-specific membrane antigen (PSMA) PET/MRI and Primovist liver MRI. Exploratory laparotomy and biopsy for confirmation of lesion and peritoneal metastasis (10).	NA

In contrast to prior reports, our patient presented with obstructive rectal symptoms, markedly elevated CEA, and an undetectable PSA, features strongly mimicking a primary colorectal malignancy. The diagnosis was particularly challenging due to limited biopsy tissue and the absence of classic prostate markers on immunohistochemistry. NGS revealed a TMPRSS2::ERG fusion identical to that present in the original prostatectomy specimen. While TMPRSS2::ERG fusions are highly characteristic of prostate carcinoma, we acknowledge that genomic alterations should not be interpreted in isolation. In this case, molecular concordance combined with histologic growth pattern and immunophenotypic findings strongly supported metastatic prostatic origin.

This case highlights the limitations of PSA and immunohistochemistry in atypical presentations and underscores the important role of molecular profiling when integrated with clinicopathologic findings in establishing the correct diagnosis. The diagnosis in this case was based on integrated clinicopathologic interpretation rather than reliance on a single genomic alteration. The predominantly submucosal growth beneath intact rectal mucosa, absence of dysplasia in the overlying epithelium, diffuse CK7 expression, focal NKX3.1 immunoreactivity, and absence of colorectal markers collectively favored metastatic prostate carcinoma over a primary colorectal malignancy. When considered alongside the molecular concordance, these findings substantially strengthened the interpretation of a prostatic origin.

CEA is not a standard biomarker for prostate cancer; however, rare cases of prostate adenocarcinoma with peritoneal or visceral metastasis have been reported to produce elevated CEA, possibly due to aberrant expression by poorly differentiated tumor cells or secondary mucinous differentiation (13, 14). In such settings, markedly elevated CEA can mimic gastrointestinal malignancy, as seen in our patient (15). The rise in CEA paralleled his disease progression, while its subsequent decline following docetaxel-based chemotherapy likely reflected treatment response and reduced tumor burden. This dynamic behavior underscores the non-specific but sometimes informative nature of CEA as a surrogate marker in atypical prostate cancer presentations.

PSMA PET imaging and NGS have transformed the landscape of cancer diagnosis and management (6, 11). NGS not only facilitates the identification of actionable genomic alterations for targeted therapy and immunotherapy but also detects hereditary mutations, such as BRCA1, BRCA2, HOXB13, and DNA repair genes, including ATM, CHEK2, and PALB2, which can inform both treatment decisions and family planning strategies (12). These advanced tools are especially valuable in diagnostically challenging cases, including those with atypical metastatic patterns or overlapping clinical features. They are also critical when conventional tumor markers like PSA remain undetectable despite disease progression. Molecular profiling and sequencing enable the precise identification of tissue-specific genetic signatures, allowing pathologists to accurately determine tumor origin even in small or anatomically difficult-to-access biopsies, as exemplified in our case.

Treatment options for prostate cancer with peritoneal metastasis remain non-standardized due to the rarity of this presentation. Management strategies in reported cases have generally followed systemic treatment paradigms used for metastatic castration-resistant prostate cancer (mCRPC). Most patients received ADT, often combined with second-line hormonal agents such as enzalutamide or abiraterone (7, 9). Chemotherapy, particularly with docetaxel, was employed in cases with aggressive progression or visceral involvement (7, 9). Radiation therapy, including SBRT, was used for local control of isolated peritoneal or nodal lesions in select cases (7). Exploratory laparoscopy served not only as a diagnostic tool but also allowed for surgical resection of accessible peritoneal nodules in some instances (7, 10).

Despite these interventions, the prognosis of patients with peritoneal metastasis remains poor, particularly in those with diffuse carcinomatosis or hepatic involvement. Our patient, initially misdiagnosed with a primary colorectal cancer, was briefly treated with FOLFOX before next-generation sequencing confirmed the prostatic origin. He responded to docetaxel-based chemotherapy, with resolution of gastrointestinal symptoms and avoidance of surgical colectomy. Clinical improvement following docetaxel and continued androgen deprivation therapy supported the interpretation of metastatic prostate carcinoma, as this response pattern would be atypical for untreated primary colorectal adenocarcinoma. This highlights the importance of individualized, timely treatment tailored by accurate diagnosis, often requiring integration of molecular diagnostics and advanced imaging to inform appropriate systemic therapy.

## Conclusion

Visceral metastases from prostate cancer are typically incurable; therefore, symptom control and preservation of quality of life remain primary goals. This case emphasizes the need for heightened awareness of rare metastatic routes such as peritoneal and rectal involvement. It also demonstrates that markedly elevated CEA and obstructive symptoms may obscure the true diagnosis, mimicking colorectal cancer. Advanced tools such as PSMA PET and next-generation sequencing are invaluable in resolving diagnostic uncertainty and guiding appropriate systemic therapy.

## Data Availability

The original contributions presented in the study are included in the article/supplementary material. Further inquiries can be directed to the corresponding author.
